# The role of IGF-2 and its variants in enhancing endothelial migration and angiogenesis

**DOI:** 10.3389/fcell.2025.1598705

**Published:** 2025-05-16

**Authors:** Lotte Alders, Elke Pirlet, Emma Gesquiere, Annelies Bronckaers

**Affiliations:** Faculty of Medicine and Life Sciences, Biomedical Research Institute (BIOMED), Hasselt University, Diepenbeek, Belgium

**Keywords:** IGF-2, IGF-2 variants, angiogenesis, endothelial cell, migration, tube formation

## Abstract

**Introduction:**

Angiogenesis, the formation of new blood vessels, is essential for physiological processes such as tissue repair as well as pathological conditions including cancer. While insulin-like growth factor 2 (IGF-2) is identified as a key regulator of angiogenesis, the contributions of its variants remain less explored.

**Methods:**

We compared the effects of wildtype IGF-2 with that of Des(1-6)IGF-2, which has lower affinity to IGF-binding proteins (IGFBPs), and Leu27IGF2, which interacts selectively with the IGF-Receptor 2. We analyzed their effect on endothelial cell migration and tube formation as well as on the secretome of endothelial cells using an antibody array. In addition, the regulatory influence of IGF-binding protein 6 (IGFBP-6) in modulating these effects was investigated. Finally, the ability of the three different variants of IGF-2 to induce blood vessel formation was studied using the chicken ‘chorioallantoic membrane’ (CAM) assay.

**Results:**

IGF-2 and Des(1-6)IGF-2 significantly promoted endothelial cell migration and tube formation *in vitro*, while also increasing blood vessel formation *in ovo*. An angiogenesis antibody array revealed that these effects were mediated through the upregulation of several angiogenic proteins, including IL-6, uPAR, and MCP-1. Interestingly, Leu27IGF-2 exhibited a weaker effect, suggesting that IGF receptor 1 and/or insulin receptor activation plays a major role in these processes. IGFBP-6 effectively inhibits IGF-2-induced effects but has no impact on Des(1-6)IGF-2, highlighting the latter’s ability to evade IGFBP-mediated inhibition due to structural modifications.

**Conclusion:**

These results suggest that Des(1-6)IGF-2 may serve as a potent pro-angiogenic agent with therapeutic potential, while IGFBP-6 could offer a strategy for suppressing pathological angiogenesis.

## 1 Introduction

Angiogenesis, the process by which new blood vessels develop from pre-existing vasculature, is essential for various physiological conditions, including embryonic development, tissue repair, and wound healing (Adair and Montani, 2010; Otrock et al., 2007; Pandya et al., 2006; Liu et al., 2023). By supplying oxygen and nutrients, it supports tissue growth and regeneration (Adair and Montani, 2010; Tahergorabi and Khazaei, 2012; Eelen et al., 2020). However, angiogenesis must be precisely balanced, as its dysregulation underlies numerous pathologies (Pandya et al., 2006; Liu et al., 2023; Tahergorabi and Khazaei, 2012; Eelen et al., 2020). Excessive angiogenesis contributes to diseases such as cancer, diabetic retinopathy, and rheumatoid arthritis (Adair and Montani, 2010; Otrock et al., 2007; Pandya et al., 2006; Liu et al., 2023; Tahergorabi and Khazaei, 2012; Carmeliet, 2003). For instance, in cancer, angiogenesis enables tumor expansion beyond the diffusion limit of 1–2 mm^3^, while also offering a vascular pathway for cancer cell dissemination (Liu et al., 2023; Eelen et al., 2020; Lee et al., 2000). Conversely, insufficient angiogenesis is a hallmark of ischemic conditions such as stroke, coronary artery disease, and chronic wounds. In these cases, inadequate vessel formation leads to impaired blood flow, tissue ischemia, and necrosis (Pandya et al., 2006; Eelen et al., 2020; Bach, 2015a). The dual role of angiogenesis—as a driver of both physiologic tissue repair and pathological progression—underscores its complexity and the need for a nuanced understanding to guide therapeutic strategies aimed at either promoting or inhibiting vascular growth (Liu et al., 2023; Eelen et al., 2020; Carmeliet, 2003; Fallah et al., 2019).

Angiogenesis primarily occurs through sprouting, a tightly regulated process involving endothelial cell (EC) activation, proliferation, migration, tube formation and vessel maturation (Adair and Montani, 2010; Eelen et al., 2020). Triggered by hypoxia or proangiogenic factors like vascular endothelial growth factor (VEGF), fibroblast growth factor-2, and insulin-like growth factors (IGFs), this process begins with EC activation and matrix metalloproteinase secretion to degrade the extracellular matrix (ECM), clearing a path for migration (Otrock et al., 2007; Fallah et al., 2019; Quintero-Fabian et al., 2019). Migrating ECs specialize into tip cells, which navigate hypoxic regions, and stalk cells, which proliferate to elongate the sprout (Eelen et al., 2020; Dallinga et al., 2018). The final stages involve tip cell fusion to form a continuous lumen, followed by pericyte recruitment and ECM deposition for vessel maturation and stability (Tahergorabi and Khazaei, 2012; Eelen et al., 2020). Given the importance of angiogenesis in tissue development, insulin-like growth factor 2 (IGF-2) emerges as a player, influencing this critical process. IGF-2 facilitates EC migration, invasion, and the formation of capillary-like networks *in vitro* and *in vivo* (Lee et al., 2000). IGF-2 is a key protein involved in growth and development and part of the broader insulin-like growth factor (IGF) system, which includes ligands (IGF-1, IGF-2, and insulin), receptors (IGF receptor 1 [IGF-R1], IGF receptor 2 [IGF-R2], and the insulin receptor [IR]), and IGF-binding proteins (IGFBPs) (Bach, 2015a; van Beijnum et al., 2017). Structurally similar to insulin, IGF-2 shares significant homology with insulin-like growth factor 1 (IGF-1), exhibiting similarities in both molecular structure and biological function. IGF-2 is primarily produced in the liver, although other tissues contribute to its expression. During fetal development, IGF-2 is highly expressed, supporting rapid tissue growth, particularly in the brain, skeleton, and muscle. Postnatally, IGF-2 levels decline, yet they remain higher than IGF-1 levels in adults. However, the precise physiological roles of IGF-2 in adulthood are less well understood (LeRoith and Roberts, 2003; Röttgering and Szuhai, 2019). IGF-2 is unique in its ability to interact with all receptors of the IGF system whereas IGF-1 and insulin lack the capacity to bind to IGF-R2. This distinction underscores the versatility of IGF-2 in mediating multiple biological effects.

IGF-R1, IR, and their hybrid form IGF-R1/IR are receptor tyrosine kinases, mediating most of the proangiogenic and mitogenic effects of IGFs by activating downstream pathways such as PI3K-Akt and RAS/MAPK, critical for EC function and survival. IGF-2 has higher affinity for, and thus preferentially binds to, IGF-R2. IGF-R2, or the cation-independent mannose 6-phosphate receptor, lacks intrinsic kinase activity and internalizes IGF-2 for lysosomal degradation, reinforcing its role as a “clearance receptor” (Bach, 2015a; van Beijnum et al., 2017; Röttgering and Szuhai, 2019; Denduluri et al., 2015). However, recent studies have revealed that IGF-R2’s functions extend beyond ligand clearance. For example, the IGF-2-IGF-R2-PLCβ2 signaling axis plays a critical role in endothelial progenitor cell recruitment, underscoring its significance in vascular biology (Maeng et al., 2009).

Insulin-like growth factor binding proteins (IGFBPs) are essential in modulating the bioavailability and activity of IGFs. To date, six distinct IGFBPs (IGFBP-1 through IGFBP-6) have been identified in the human body. These proteins not only extend the half-life of IGFs but also modulate their tissue distribution. IGFBPs, traditionally thought to inhibit IGF actions by blocking receptor binding, are now understood to have a more complex context-dependent role, either enhancing or inhibiting IGF effects (Bach, 2018; Clemmons, 1997). For instance, IGFBP-2 promotes angiogenesis by enhancing IGF-2-mediated VEGF transcription (Yau et al., 2015). Conversely, IGFBP-4 and IGFBP-6 inhibit IGF activity by sequestering the ligands in binary or ternary complexes, thereby limiting their bioavailability and preventing IGFs from crossing capillary walls (Bach, 2018; Clemmons, 1997).

Synthetic IGF-2 variants, such as Des(1-6)IGF-2, which lacks the N-terminal hexapeptide, retain the capacity to bind the IGF-2 receptors, but exhibit reduced interaction with IGFBPs. This modification bypasses the sequestration effect imposed by IGFBPs, thereby enhancing the bioavailability and signaling potency (Francis et al., 1993). In contrast, another variant, Leu27IGF-2, features a leucine substitution at position 27, inducing a selective binding profile for IGF-R2 without affecting IGFBP binding. This enables focused investigation of IGF-R2-specific effects on angiogenesis and EC function (Beukers et al., 1991; Chen R. J. et al., 2009).

While numerous studies have highlighted the role of IGF-2 in blood vessel formation, the specific effects of structurally distinct variants—such as Des(1-6)IGF-2 and Leu27IGF-2—on EC behavior remain underexplored. Notably, this study is, to our knowledge, the first to investigate the role of Des(1-6)IGF-2 in the context of angiogenesis. In this study, we investigated the presence of different IGF-2 receptors on ECs, which provided the foundation for elucidating the effects of IGF-2, Des(1-6)IGF-2, and Leu27IGF-2 on EC migration and tube formation—critical steps in the angiogenesis process. Additionally, the influence of IGFBPs on these processes was investigated. Utilizing an antibody array and ELISA, the modulation of angiogenic protein secretion by ECs in the presence and absence of IGFBP-6 was explored for IGF-2 and Des(1-6)IGF-2. To gain further insights into the *in ovo* angiogenic properties of IGF-2 and its variants, the chicken chorioallantoic membrane (CAM) assay was carried out. Together, these findings will provide new insights into how different IGF-2 variants may differentially regulate angiogenesis, laying a foundation for more targeted therapeutic strategies aimed at promoting or inhibiting angiogenesis.

## 2 Materials and methods

### 2.1 HMEC-1 cell culture

The human microvascular endothelial cell-1 (HMEC-1) line was procured from the Centers for Disease Control and Prevention [CLS Cat# 304064, RRID:CVCL_0307, Atlanta, GA, United States, (Ades et al., 1992)]. Cells were cultured in standard HMEC-1 growth medium comprising MCDB 131 medium (10372-019, Gibco, Paisley, United Kingdom) supplemented with 10% heat-inactivated fetal bovine serum (FBS, s181B-500, Biowest, Nuaillé, France), 100 U/ml Penicillin and 100 μg/ml Streptomycin (1% P/S, P4333, Sigma-Aldrich, Saint Louis, MO, United States), 10 mM L-glutamine (G7513, Sigma-Aldrich, Saint Louis, MO, United States), 10 ng/mL human epidermal growth factor (EGF, PGH0311L, Gibco, Paisley, United Kingdom) and 1 μg/ml hydrocortisone (A16292.03, Alfa Aesar, Thermo Fisher Scientific, Haverhill, MA, United States). Cultures were maintained at 37°C in a humidified incubator with 5% CO_2_. The medium was refreshed every two to 3 days to remove metabolic waste and replenish nutrients. Cellular morphology and confluency were monitored daily using an inverted phase-contrast microscope (Nikon Eclipse Ts2, RRID:SCR_025716, Nikon Europe, Amstelveen, Netherlands). Upon reaching 80% cell confluency, cells were passaged using 0.05% trypsin-EDTA (T3924, Sigma-Aldrich, Saint Louis, MO, United States), as detachment agent. HMEC-1 from passages four to eleven were used for experiments. The HMEC-1 cell line was routinely tested for *mycoplasma* contamination and were confirmed to be mycoplasma-free.

### 2.2 Immunocytochemistry – presence of IGF receptors

HMEC-1 were seeded onto glass coverslips at a density of 4 × 10^4^ cells per well and allowed to adhere overnight. Following attachment, cells were fixed with 4% paraformaldehyde (PFA) for 15 min at room temperature (RT) to preserve cellular morphology. To minimize non-specific antibody binding, cells were blocked with 100% protein block (PB; X0909, Agilent/Dako, Santa Clara, CA, United States) for 20 min at RT. Primary antibodies (details provided in Table 1) were diluted in 10% PB and incubated overnight at 4°C. Subsequently, cells were incubated with secondary antibodies (details provided in Table 1) and Hoechst 33342 nuclear stain (1:5,000 dilution, H-3570, RRID:AB_3675235, Invitrogen – Thermo Fisher Scientific, Waltham, MA, United States) in 10% PB for one hour at RT in the dark. After staining, coverslips were mounted onto glass slides using anti-fade mounting medium (Fluoromount-G, 00-4959-52, Invitrogen – Thermo Fisher Scientific, Waltham, MA, United States) to preserve fluorescence. Imaging was performed using a Leica DM2000 LED fluorescence microscope (RRID:SCR_020223, Leica Microsystems, Wetzlar, Germany) equipped with Leica Application Suite X software (RRID:SCR_013673, Leica Microsystems, Wetzlar, Germany).

**TABLE 1:** Primary and secondary antibodies.

Primary Antibodies			Secondary Antibodies		
Target	Dilution	Reference details	Target	Dilution	Reference details
Goat anti-IGF-R1	1:20	NB300- 514 (RRID:AB_2139105) Novus Biologicals (Bio-Techne, Minneapolis, MN, United States)	Donkey anti-goat Alexa Fluor^TM^ 555	1:500	A21432 (RRID:AB_141788) Life Technologies (Carlsbad, CA, United States)
Mouse anti-IGF-R2	1:100	AF-305- NA (RRID:AB_354457) R&D systems (Bio-Techne, Minneapolis, MN, United States)	Donkey anti-mouse Alexa Fluor^TM^ 555	1:500	A31570 (RRID:AB_2536180) Life Technologies (Carlsbad, CA, United States)
Rabbit anti-IR	1:100	NBP2- 16970 (RRID:AB_3263926) Novus Biologicals (Bio-Techne, Minneapolis, MN, United States)	Goat anti-rabbit Alexa Fluor^TM^ 555	1:500	A21430 (RRID:AB_10374475) Life Technologies (Carlsbad, CA, United States)

IGF-R1, insulin-like growth factor receptor type 1; IGF-R2: insulin-like growth factor receptor type 2; IR: insulin receptor.

### 2.3 Chemotaxis transwell migration assay

A total of 1.5 × 10^3^ HMEC-1 were seeded into the inserts of an Incucyte ClearView 96-well Chemotaxis Plate (4582, Sartorius, Göttingen, Germany) in alpha-MEM medium (MEM-XRXA, Capricorn Scientific, Ebsdorfergrund, Germany) supplemented with 0.05% FBS, 2 mM L-glutamine and 1% P/S. The cells were allowed to settle for 30 min. Test conditions included 100 ng/mL IGF-2 (FU100, GroPep Bioreagents, Thebarton, Australia), Des(1-6)IGF-2 (MU100, GroPep Bioreagents, Thebarton, Australia) or Leu27IGF- (TU100, GroPep Bioreagents, Thebarton, Australia) either alone or in combination with 1,000 ng/mL IGFBP-6 (876-B6-025, Bio-Techne, Minneapolis, MN, United States) were assessed. α-MEM with 0.05% FBS served as the negative control, while α-MEM with 10% FBS was used as the positive control. Test conditions were added to the bottom wells of the chemotaxis plate, and inserts containing the seeded cells were carefully placed into the corresponding wells. Plates were incubated at 37°C for 5 days, while cell migration was continuously monitored using the Incucyte S3 Live-Cell Analysis System (RRID:SCR_023147, Sartorius, Göttingen, Germany), with images captured every two hours. Migration was quantified using the Incucyte “chemotaxis” analysis tool (No. 9600-0015, RRID:SCR_017316, Sartorius, Göttingen, Germany), which measured the average cell area (µm^2^) on the underside of the insert membrane, representing cells that migrated successfully in response to the test conditions.

### 2.4 Scratch wound healing assay

HMEC-1 were seeded into an Incucyte 96-well ImageLock plate (BA-04856, Sartorius, Göttingen, Germany) at a density of 35,000 cells per well in standard HMEC-1 growth medium. The cells were cultured for 24 h to allow the formation of a confluent monolayer. Subsequently, a uniform scratch was created in each well using the 96-well WoundMaker tool (4563, Sartorius, Göttingen, Germany) according to the manufacturer’s instructions. After washing, test conditions were diluted using HMEC-1 scratch medium, which consisted of MCDB 131 medium supplemented with 0.05% FBS, 2 mM L-glutamine and 1% P/S. The test conditions included treatment with IGF-2, Des(1–6)IGF-2 or Leu27IGF-2 at a concentration of 100 ng/mL either alone or in combination with 1,000 ng/mL IGFBP-6. HMEC-1 scratch medium served as the negative control, while HMEC-1 scratch medium with 10% FBS was added as the positive control. The cells were incubated at 37°C for 48 h. Images of the wound area were captured every two hours using the Incucyte S3 Live-Cell Analysis system. Migration into the wound area was assessed using the Incucyte software’s “scratch wound” tool (9600-0012, Sartorius, Göttingen, Germany), which quantified the progression of healing as relative wound density (RWD). This integrated metric represents the density of cells in the wound area relative to the density of the surrounding monolayer, calculated using the following equation: $\%RWD\left(t\right)=100*\;\frac{\left(w\left(t\right)-w\left(0\right)\right)}{\left(c\left(t\right)-w\left(0\right)\right)}$ with *w(t)* representing density of the wound region at time (t) and *c(t)* representing the density of cell region at time (t).

### 2.5 Tube formation assay

A 10 μL volume of ice-cold growth factor reduced Matrigel (356231, Corning, Bedford, Massachusetts, United States) was carefully added to each well of an angiogenesis μ-slide (81506, Ibidi, Gräfelfing, Germany) and allowed to polymerize at 37°C for 30 min. Cells and test conditions were diluted in α-MEM supplemented with 2 mM L-glutamine and 1% P/S. This medium served as negative control while this medium supplemented with 10% FBS served as positive control. HMEC-1 were seeded at a density of 10,000 cells per well for each condition. The experimental conditions tested included treatment with 100 ng/mL of IGF-2, Des(1–6)IGF-2 or Leu27IGF-2 alone or in combination with 1,000 ng/mL IGFBP-6. After eight hours of incubation, images of the capillary-like structures were captured using an inverted phase-contrast light microscope with a ×4 objective. Tube formation was quantified using the Gilles Carpentier Angiogenesis Analyzer (Carpentier et al., 2020) in Fiji ImageJ software [RRID:SCR_002285, (Schindelin et al., 2012)]. From the analyzed parameters, the number of nodes (how many times segments connect or branch) and the total tube length (lengths of all segments) were selected for presentation.

### 2.6 Human angiogenesis antibody array

HMEC-1 were cultured at a density of 2 × 10^5^ cells in standard HMEC-1 growth medium for 24 h. After this incubation period, the medium was replaced with test conditions diluted in MCDB 131 medium supplemented with 0.05% FBS, 2 mM L-glutamine and 1% P/S. The test conditions applied to the cells included the addition of 100 ng/mL IGF-2 and Des(1-6)IGF-2 alone or combined with 1,000 ng/mL IGFBP-6. After 48 h of treatment, the conditioned medium (CM) was collected, and the Human Angiogenesis Antibody Array (AAH-ANG-1000-2, RayBiotech LucernaChem, Luzern, Switzerland) was performed as per the manufacturer’s instructions. The signal from the array was visualized using an Amersham Imager 680 (GE Healthcare Life Sciences, Chicago, IL, United States), and the results were analyzed using the Gilles Carpentier Protein Array Analyzer software (Carpentier, 2010) in Fiji ImageJ (Schindelin et al., 2012). The integrated pixel densities for each protein were calculated by subtracting the background signal and normalizing to the positive reference spots. The results are represented in a heat map format.

### 2.7 Enzyme-linked immunosorbent assay (ELISA)

To validate the semi-quantitative findings obtained from the Angiogenesis Antibody array, concentrations of interleukin-6 (IL-6, 430504, BioLegend, San Diego, CA, United States), urokinase plasminogen activator (uPAR, 448404, BioLegend, San Diego, California, United States), and monocyte chemoattractant protein-1 (MCP-1, 438804, BioLegend, San Diego, CA, United States) were measured in the CM collected from HMEC-1 following 48 h of treatment with the test conditions described in Section 2.6 Human Angiogenesis Antibody Array. ELISAs were carried out according to the respective manufacturer’s guidelines for each target. Briefly, wells were coated with the appropriate capture antibody overnight at 4°C. The following day, wells were rinsed and blocked with a blocking solution for one hour at RT to block non-specific binding and reduce background signal. Subsequently, the standards and samples were added and incubated for two hours at RT. After removal of unbound material, the detection antibody was incubated for one hour at RT followed by a 30-min incubation of Avidin-HRP. After a final wash step, substrate solution was added, and incubated in the dark for 15–25 min (depending on target). The reactions were terminated using stop solution, and the absorbance at 450 nm was measured using the CLARIOstar Plus plate reader (RRID:SCR_026330, BMG Labtech, Ortenberg, Germany). The absorbance values of the standard dilution series, with known concentrations, were used to determine the concentrations of the samples based on their absorbance readings.

### 2.8 Chorioallantoic membrane (CAM) assay

The angiogenic capacity of IGF-2 and variants were tested in an *in ovo* CAM assay. Fertilised chicken eggs (Gallus Gallus, RRID:NCBITaxon_9031, Wyverkens poultry farm, Halle, Belgium) were incubated at 37°C and 50% humidity. On embryonic day 3 (E3), 3–4 mL of albumin was withdrawn using a syringe to detach the CAM from the eggshell. The eggs were then returned to the incubator. At E9, a small window (∼1.5 cm^2^) was created in the eggshell to expose the CAM. Absorbent gelatin sponges (ZHG805001, SMI AG, Sankt Vith, Belgium) were cut into ∼1 mm^3^ pieces and placed onto the CAM. A volume of 5 µL of test solution was applied to each sponge. The test conditions included 5 ng IGF-2, Des(1-6)IGF-2 or Leu27IGF-2. HCl at a concentration of 0.05 µM (also 5 µl) served as negative control. The window was sealed with adhesive tape and the eggs were returned to the incubator for 48 h. On E11, the CAMs were excised using scissors and photographed (Handycam HDR-XR350VE, Sony, Minato, Tokyo, Japan). Angiogenesis was assessed by counting blood vessels intersecting a concentric circle with a radius of 3 mm, which was digitally added onto the CAM images, with the center of the circle on the sponge. Vessel counting was performed blindly by two independent investigators and the average of these counts was calculated for each egg.

### 2.9 Statistical analysis

All data are reported as mean ± standard error of mean (SEM). Statistical analyses were performed using GraphPad Prism version 10.1.1 (RRID:SCR_002798, GraphPad Software, San Diego, CA, United States). Outliers were identified and removed using Grubb’s test. The normality of the data was assessed using the Shapiro-Wilk test. For comparisons between the negative control and the variants, statistical analyses were performed depending on the presence of missing data and repeated measures. If no data were missing, a one-way ANOVA with Dunnett’s multiple comparisons test was used. In cases where missing data were present, a mixed-effects analysis with Dunnett’s test was applied. For repeated measures data, a repeated measures ANOVA with Dunnett’s test was conducted. When comparing IGF-2 or its variant Des(1-6)IGF-2 to their IGFBP-6 combination counterparts, a one-way ANOVA with Sidak’s multiple comparisons test was performed for complete datasets. If missing data were present, a mixed-effects analysis with Sidak’s test was used. For repeated measures data, a repeated measures ANOVA with Sidak’s test was applied. The number of experimental replicates and the specific statistical tests used are detailed in the figure legends.

## 3 Results

### 3.1 HMEC-1 express all three IGF-2 receptors

**FIGURE 1:**
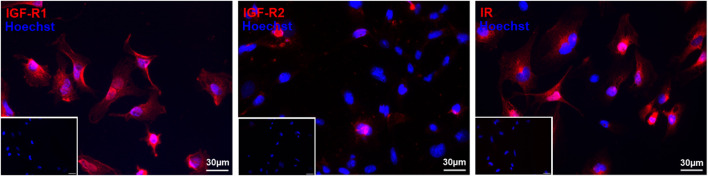
Immunofluorescent staining of IGF-R1, IGF-R2, and IR confirms the presence of all three receptors in HMEC-1. Cells were stained with primary antibodies for IGF-R1, IGF-R2 and IR (red) and nuclei are counterstained with Hoechst (blue). Representative immunofluorescent images confirm the widespread expression of IGF-R1 and IR, while IGF-R2 is detected in only a limited number of cells. Inserts display the corresponding negative controls. Scale bar: 30 µm. IGF-R1, insulin-like growth factor – receptor 1; IGF-R2, insulin like growth factor – receptor 2; IR, insulin receptor; HMEC-1, human microvascular endothelial cells.

To determine whether HMEC-1 have the capacity to respond to IGF-2 stimulation, it was essential to first study the presence of its receptors. Immunocytochemistry confirmed the expression of all three receptors: IGF-R1, IGF-R2 and IR (Figure 1). The majority of cells were positive for IGF-R1 and IR, while only a few cells showed staining for IGF-R2. The confirmed presence of all receptors suggests that HMEC-1 are capable of interacting with IGF-2, providing a foundation for further investigating its functional effects.

### 3.2 IGF-2 and its variants enhance endothelial cell migration with IGFBP-6 negating the effect of IGF-2 but not Des(1-6)IGF-2

**FIGURE 2:**
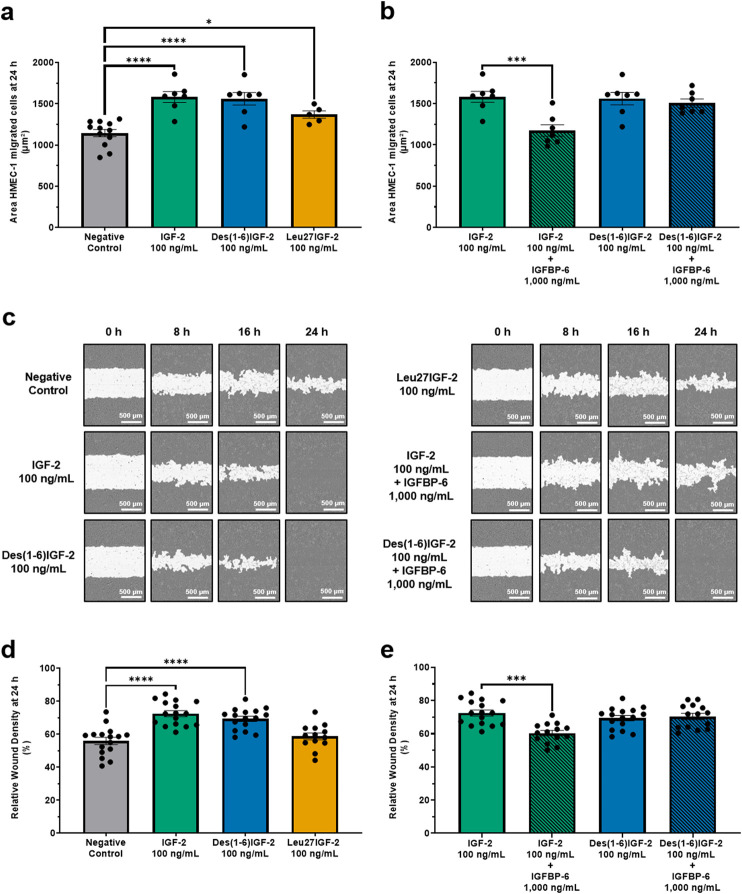
IGF-2 and its variants enhance HMEC-1 migration. **(a,b)** Chemotactic migration was assessed using a transwell migration assay with the IncuCyte® S3 Live-Cell Analysis System. **(a)** At 24 h, all variants significantly increased HMEC-1 migration compared to the negative control (n = 7-12, one-way ANOVA with Dunnett’s multiple comparisons test). **(b)** The addition of IGFBP-6 to IGF-2 significantly reduced IGF-2-induced migration, while IGFBP-6 had no effect on the migratory response to Des(1-6)IGF-2 (n = 7, one-way ANOVA with Sidak’s multiple comparisons test). **(c–e)** Results of the scratch wound healing assay, as monitored using the IncuCyte® system. **(c)** Representative images of the wound area (white) over time. Scale bar: 500 µm. **(d)** Migration is expressed as relative wound density (cell density in the wound area relative to the cell density outside the wound area). Cells were treated with 100 ng/mL IGF-2 or Des(1-6)IGF-2, which significantly enhanced wound closure at 24 h, while Leu27IGF-2 had no significant effect (n = 16, mixed-effects analysis with Dunnett’s multiple comparisons test). **(e)** IGFBP-6 showed an inhibitory effect on IGF-2-induced migration, while Des(1-6)IGF-2-induced migration remained unaffected (n = 14-16, mixed-effects analysis with Sidak’s multiple comparisons test). Data are presented as mean ± SEM. *p < 0.05, ***p < 0.001, ****p < 0.0001. HMEC-1, human microvascular endothelial cells; IGF-2, insulin-like growth factor 2; IGFBP-6, IGF-binding protein 6.

EC migration is a critical step in the process of angiogenesis, enabling the formation of new blood vessels by allowing ECs to move into areas in need of tissue remodeling. The effect of IGF-2 and its variants, Des(1-6)IGF-2 and Leu27IGF-2 on HMEC-1 migration was assessed using both the transwell migration assay and the scratch wound healing assay. In the chemotaxis transwell migration assay, 100 ng/mL of IGF-2, Des(1-6)IGF-2 and Leu27IGF-2 significantly increased HMEC-1 migration by 38% (1,582 µm^2^ ± 66 μm^2^, p < 0.0001), 36% (1,561 µm^2^ ± 75 μm^2^, p < 0.0001) and 20% (1,368 µm^2^ ± 45 μm^2^, p = 0.0438) respectively, compared to the negative control (1,144 µm^2^ ± 45 μm^2^) after 24 h (Figure 2a). The addition of IGFBP-6 to IGF-2 significantly reduced IGF-2-induced migration by almost 26%, bringing it back to negative control levels (1,582 µm^2^ ± 66 μm^2^ vs. 1,174 µm^2^ ± 70 μm^2^, p = 0.0004), while IGFBP-6 had no effect on the stimulating migratory effect of Des(1-6)IGF-2 (1,561 µm^2^ ± 75 μm^2^ vs. 1,507 µm^2^ ± 49 μm^2^, p = 0.8123) (Figure 2b). Similar results were observed in the scratch wound healing assay (Figure 2c), where IGF-2 significantly enhanced wound closure at 24 h by increasing relative wound density by 30% compared to the negative control (56% ± 1.9% vs. 73% ± 1.8%, p < 0.0001). Des(1-6)IGF-2 also promoted a significant 23% increase in relative wound density (56% ± 1.9% vs. 69% ± 1.6%, p < 0.0001). In contrast, Leu27IGF-2 did not induce wound closure compared to control conditions (56% ± 1.9% vs. 59% ± 2.1%, p = 0.2174) (Figure 2d). Combining IGFBP-6 with IGF-2, significantly reduced the wound density with 18% compared to IGF-2 alone (73% ± 1.8% vs. 60% ± 1.6%, p = 0.0002), but it had no effect on Des(1-6)IGF-2-induced migration (69% ± 1.6% vs. 70% ± 1.9%, p = 0.6537) (Figure 2e).

**FIGURE 3:**
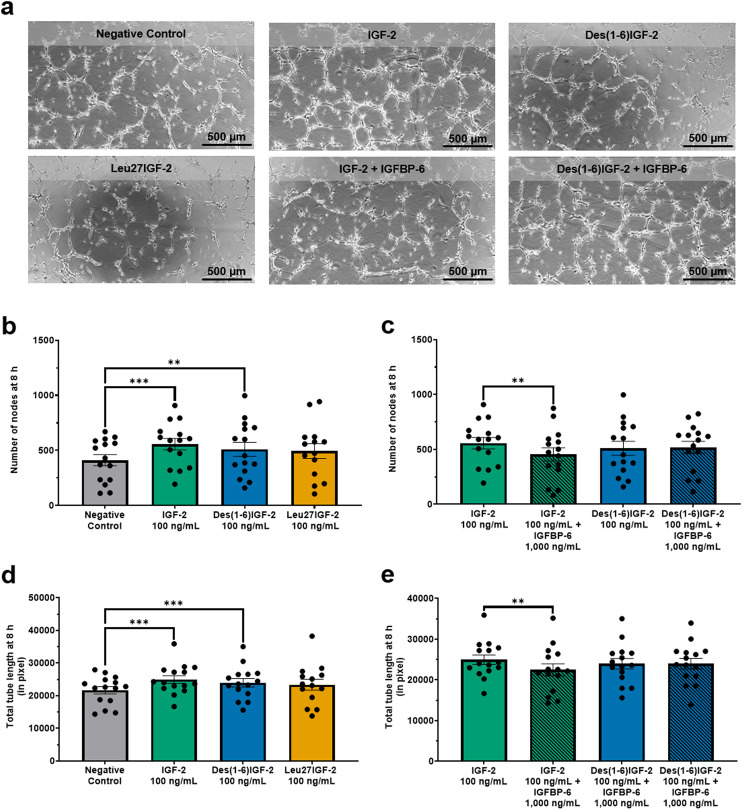
IGF-2 and Des(1-6)IGF-2 promote endothelial tube formation. HMEC-1were seeded on Matrigel-coated angiogenesis μ-slides and treated with 100 ng/mL IGF-2, Des(1-6)IGF-2, or Leu27IGF-2. IGF-2 and Des(1-6)IGF-2 were also tested combined with 1,000 ng/mL IGFBP-6. After 8 h, tube formation was quantified and two key parameters were analyzed: number of nodes, indicating network complexity, and total tube length (in pixel, measuring overall network size. **(a)** Representative images of tube formation at 8 h for all conditions. Scale bar: 500 µm. **(b,d)** IGF-2 and Des(1-6)IGF-2 significantly increased node formation **(b)** and total tube length **(d)**, while Leu27IGF-2 had no effect (n = 15–20). **(c,e)** IGFBP-6 inhibited IGF-2-induced increases in all parameters but did not affect Des(1-6)IGF-2 (n = 15–20). Mixed-effects analysis with Dunnett’s multiple comparisons test was used to compare IGF-2 and variants to the negative control. For comparing IGF-2 and Des(1-6)IGF-2 with their combination with IGFP-6, repeated measures ANOVA with Sidak’s multiple comparisons test was used. Data are presented as mean ± SEM. **p < 0.01, ***p < 0.001. HMEC-1: human microvascular endothelial cells; IGF-2, insulin-like growth factor 2; IGFBP-6, IGF-binding protein 6.

Next, the tube formation assay, which evaluates the ability of ECs to form capillary-like structures was performed (Figure 3). Two key parameters were analyzed: number of nodes and total length. These parameters provide insight into the extent, complexity, and overall length of tube formation. For the number of nodes, IGF-2 and Des(1-6)IGF-2 significantly increased node formation by 36% (557 ± 52, p = 0.0002) and 24% (510 ± 64, p = 0.048), respectively, compared to the negative control (411 ± 50) (Figure 3b). The addition of IGFBP-6 significantly reduced IGF-2-induced node formation by 19% (557 ± 52 vs. 452 ± 60, p = 0.0090) but had no effect on Des(1-6)IGF-2-induced formation (510 ± 64 vs. 518 ± 56, p = 0.9732) (Figure 3c). A similar trend was observed for the overall size of the network including all branches and segments. For total tube length, IGF-2 and Des(1-6)IGF-2 significantly enhanced tube formation by 15% (21,675 pixel ± 1,129 pixel vs. 24,968 pixel ± 1,172 pixel, p = 0.0006) and 11% (21,675 pixel ± 1,129 pixel vs. 23,964 pixel ± 1,287 pixel, p = 0.0009), respectively (Figure 3d). IGFBP-6 significantly suppressed IGF-2-induced tube formation capacity by 10% (24,968 pixel ± 1,172 pixel vs. 22,475 pixel ± 1,472 pixel, p = 0.0048) but did not impact Des(1-6)IGF-2-induced tube formation (23,964 pixel ±1,287 pixel vs. 23,987 pixel ± 1,289 pixel, p = 0.9995) (Figure 3e). Furthermore, Leu27IGF-2 had no effect on the number of nodes and the total tube length.

### 3.3 IGF-2 and Des(1-6)IGF-2 induce the expression of various angiogenic factors *in vitro* with IGFBP-6 inhibiting the effect of IGF-2 but not of Des(1-6)IGF-2

**FIGURE 4:**
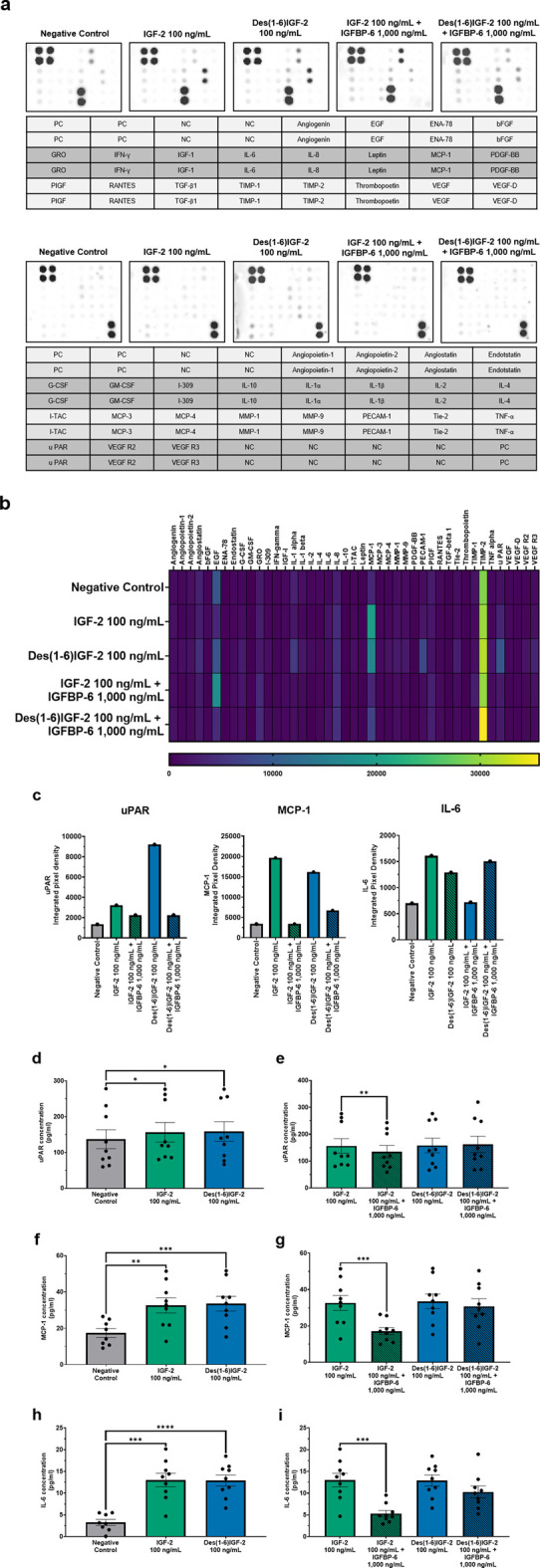
Angiogenic protein profiles of HMEC-1 cells treated with IGF-2 or Des(1-6)IGF-2 alone or in combination with IGFBP-6. A screening for 43 human angiogenic factors was performed on conditioned medium collected from HMEC-1 cells treated with medium, 100 ng/mL IGF-2, 100 ng/mL Des(1-6)IGF-2, 100 ng/mL IGF-2 + 1,000 ng/mL IGFBP-6, or 100 ng/mL Des(1-6)IGF-2 + 1,000 ng/mL IGFBP-6. **(a)** Antibody blots for each condition is shown with the corresponding protein location in the layout (n = 1). The 43 proteins were divided over two separate blots. **(b)** Heatmap displaying pixel density is expressed for all detected proteins. **(c)** Pixel densities of three selected targets—uPAR, MCP-1 and IL-6 are shown. **(d–i)** Elisa validation and quantification (pg/ml) of uPAR (n = 9) **(d,e)**, MCP-1 (n = 8-9) **(f,g)** and IL-6 (n = 8-9) **(h,i)** in conditioned medium. Concentration of all targets increase upon IGF-2 or Des(1-6)IGF-2 stimulation. IGFBP-6 inhibits effects of IGF-2 but not Des(1-6)IGF-2. Statistical analysis for ELISA: comparisons between negative control and IGF-2 or Des(1-6)IGF-2 were performed using repeated measures ANOVA with Holm-Sidak multiple comparisons test, while comparisons between IGF-2 vs. IGF-2 + IGFBP-6 and Des(1-6)IGF-2 vs. Des(1-6)IGF-2 + IGFBP-6 were analyzed using repeated measures ANOVA with Sidak’s multiple comparisons test. Data are presented as mean ± SEM. *p < 0.05, **p < 0.01, ***p < 0.001, ****p < 0.0001. HMEC-1: human microvascular endothelial cells; NC, negative control; PC, positive control; IGF-2, insulin-like growth factor 2; IGFBP-6, IGF-binding protein 6.

In order to study the influence of IGF-2 and variants on the angiogenic profile of HMEC-1, an angiogenesis antibody array for 43 proteins was performed on the CM of HMEC-1 cells treated with IGF-2 or Des(1-6)IGF-2 alone or together with IGFBP-6. A wide range of angiogenic factors could be detected as shown in the representative blots (Figure 4a). A heatmap summarizing the relative expression levels of all detected proteins is presented in Figure 4b. Several proteins exhibited differential expression upon IGF-2 or Des(1-6)IGF-2 treatment compared to the control condition (Figure 4c). The impact of IGF-2, Des(1-6)IGF-2 alone and in combination with IGFBP-6, on angiogenic protein secretion was validated using ELISA for three selected targets: uPAR, MCP-1, and IL-6 (Figures 4d–i). For uPAR, IGF-2 and Des(1-6)IGF-2 significantly increased secretion levels by 14% and 15%, respectively, compared to the control (137 pg/ml ± 26.6 pg/ml vs. 156 pg/ml ± 27.1 pg/ml, p = 0.0166; 133 pg/ml ± 26.6 pg/ml vs. 158 pg/ml ± 27.4 pg/ml, p = 0.194). The addition of IGFBP-6 significantly reduced IGF-2-induced uPAR secretion by 13% (156 pg/ml ± 27.1 pg/ml vs. 135 pg/ml ± 22.9 pg/ml, p = 0.0077) but had no significant effect on Des(1-6)IGF-2 (Figures 4d,e). A similar trend was observed for MCP-1, where IGF-2 and Des(1-6)IGF-2 significantly increased MCP-1 levels by 87% and 93%, respectively (17.4 pg/ml ± 2.4 pg/ml vs. 32.6 pg/ml ± 4.2 pg/ml, p = 0.0010; 17.4 pg/ml ± 2.4 pg/ml vs. 33.5 pg/ml ± 4.0 pg/ml, p = 0.0007). IGFBP-6 significantly inhibited IGF-2-induced MCP-1 secretion by 48% (32.6 pg/ml ± 4.2 pg/ml vs. 17.1 pg/ml ± 2.0 pg/ml, p = 0.0003), whereas Des(1-6)IGF-2-induced MCP-1 secretion did not drop (Figures 4f,g). For IL-6, both IGF-2 and Des(1-6)IGF-2 significantly increased secretion by 294% and 291%, respectively (3.3 pg/ml ± 0.7 pg/ml vs. 13.0 pg/ml ± 1.5 pg/ml, p = 0.0002; 3.3 pg/ml ± 0.7 pg/ml vs. 12.9 pg/ml ± 1.3 pg/mL, p < 0.0001). IGFBP-6 significantly decreased IGF-2-induced IL-6 secretion by 59% (13.0 pg/ml ± 1.5 pg/ml vs. 5.3 pg/ml ± 0.7 pg/ml, p = 0.0003) while there was no significant effect on Des(1-6)IGF-2-induced IL-6 secretion (Figures 4h,i).

### 3.4 IGF-2 and Des(1-6)IGF-2 enhance blood vessel formation in the CAM assay

**FIGURE 5:**
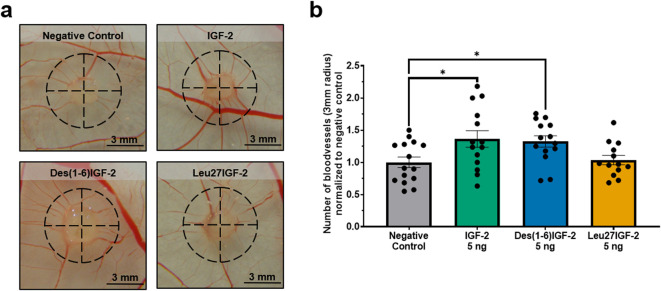
*In ovo* angiogenic effect of IGF-2 and Des(1-6)IGF-2 on the chorioallantoic membrane (CAM). Sponges containing vehicle, 5 ng of IGF-2, 5 Des(1-6)IGF-2, or Leu27IGF-2 were placed on the CAM of chicken embryos at embryonic day 9. After 48 h, CAMs were photographed and the number of blood vessels intersecting a 3 mm radius around each sponge was manually counted (n = 13–15 eggs for each treatment, from 3 independent CAM experiments). **(a)** Representative images of the CAM with the analyzed area indicated by a dashed black circle. Scale bars represent 3 mm. **(b)** Quantification of the number of blood vessels intersecting the 3 mm radius, normalized to the negative control. Data are expressed as mean ± S.E.M. *p < 0.05 compared to the negative control, as determined by one-way ANOVA followed by Dunnett’s post-hoc test. CAM, chicken chorioallantoic membrane; IGF-2, insulin-like growth factor 2; IGFBP-6, IGF-binding protein 6.

Finally, the paracrine potential of IGF-2 and its variants to stimulate *in ovo* formation of functional blood vessels was assessed in the CAM assay. Gelatin sponges were placed on the CAM and either vehicle control, IGF-2, Des(1-6)IGF-2, or Leu27IGF-2 were applied to the sponge and 48 h later the number of blood vessels within a 3 mm radius around the sponge was quantified (Figure 5a). IGF-2 significantly increased the number of vessels compared to the negative control (normalized to 1.00 ± 0.08) by 36% (1.36 ± 0.13, p = 0.0238). Similarly, Des(1-6)IGF-2 significantly enhanced vessel formation, resulting in a 32% increase (1.32 ± 0.09, p = 0.0491) (Figure 5b). In contrast, Leu27IGF-2 did not significantly alter vessel density (1.04 ± 0.07, p = 0.9881).

## 4 Discussion and conclusion

Angiogenesis is tightly regulated by a balance of pro- and anti-angiogenic factors, with IGF-2 emerging as a potential regulator (Pandya et al., 2006; Liu et al., 2023; Tahergorabi and Khazaei, 2012; Eelen et al., 2020; Lee et al., 2000). Despite the established link between IGF-2 and vascular development, the intricacies of its receptor interactions, and the influence of IGFBP’s on endothelial cell (EC) behavior are not fully elaborated. With this study, we aimed to elucidate the effects IGF-2 and its variants on angiogenesis, including IGFBP-mediated modulation. Furthermore, to our knowledge, we are the first to investigate the role of Des(1-6)IGF-2 in this context and to map the EC secretome following incubation with IGF-2 or Des(1-6)IGF-2. Our findings shed light on the nuanced roles of IGF-2 variants in EC behavior, advancing our knowledge of IGF-2 signaling in vascular biology and paving the way for more targeted therapeutic strategies in angiogenesis-related diseases.

We first assessed the presence of IGF-2 receptors in HMEC-1 using immunocytochemistry to determine their responsiveness to IGF-2. All three IGF-2 receptors—IGF-R1, IGF-R2, and IR—were detected, though the proportion of cells expressing each receptor varied. This suggests constitutive receptor expression in HMEC-1 under normal conditions, enabling ECs to efficiently respond to IGF-2 stimulation. This is in line with the well-established role of IGF-2 as a growth factor that activates key signaling pathways, such as PI3K-Akt and RAS/MAPK, which regulate EC proliferation, migration, and survival (Bach, 2015a; Hakuno and Takahashi, 2018). Our findings are consistent with earlier studies, dating back to the 1980s, that identified insulin and IGF receptors on ECs, implicating them in vascular biology (Bar et al., 1981; Frank and Pardridge, 1981; Banskota et al., 1986). Later studies by Nitert et al. confirmed the presence of IGF-R1, IGF-R2, and IR in human umbilical vein ECs (HUVECs) through gene expression analysis, reporting that HUVECs exhibit higher levels of IGF-R1 compared to IR (Nitert et al., 2005). Similarly, Chiselita et al. analyzed human coronary artery ECs and found a comparable expression pattern, with IGF-R1 being more highly expressed than IR (Chisalita et al., 2006). In contrast to the widespread expression of IGF-R1 and IR, IGF-R2 was detected in only a small subset of cells, suggesting that its expression may be more tightly regulated depending on cellular needs. Consistent with our findings, Volpert et al. also reported IGF-R2 expression in bovine adrenal capillary ECs (Volpert et al., 1996). As IGF-R2 primarily mediates IGF-2 degradation, its variable expression may modulate IGF-2 availability and downstream signaling (Bach, 2015a). This suggests that under normal conditions, IGF-2 signaling regulation in HMEC-1 cells may depend more on activation than on degradation. While our data confirm receptor presence, it does not allow quantification of relative abundance. Additionally, it remains unclear how receptor expression changes under pathological conditions or IGF-2 treatment—an important avenue for future studies to refine our understanding of IGF-2 signaling in angiogenesis.

Next, we investigated the functional impact of IGF-2 on EC migration, which plays a vital role in angiogenesis (Lamalice et al., 2007). Our findings indicate that IGF-2 and its variants, Des(1-6)IGF-2 and Leu27IGF-2, significantly enhance cell migration as demonstrated by the transwell migration assay. IGF-2 and Des(1-6)IGF-2 elicited comparable increases in migration of 38% and 36% respectively, whereas Leu27IGF-2 exhibited a more modest increase of 20%. The scratch wound healing assay corroborated these findings, with IGF-2 and Des(1-6)IGF-2 both significantly enhancing wound closure at 24 h. IGF-2 increased relative wound density by 30%, while Des(1-6)IGF-2 induced a 23% increase. In contrast, Leu27IGF-2 did not improve the relative wound density. These findings suggest that IGF-2 and Des(1-6)IGF-2 possess strong pro-migratory properties, driving the migration in HMEC-1 cells, whereas Leu27IGF-2 appears less potent in this context. Numerous studies have underscored the pivotal role of IGF-2 in promoting cellular migration across diverse biological contexts. In ovine trophectoderm cells, a concentration of 50 ng/mL IGF-2 enhanced migration by approximately 197% (Kim et al., 2008). Similarly, a study by Xu et al. demonstrated that IGF-2 secreted by cancer cells can directly enhance fibroblast migration by more than 200%. Notably, this effect was abolished with an IGF-2 neutralizing antibody (Xu et al., 2017). Conversely, Chen et al. reported that while 100 ng/mL IGF-2 did not enhance the migration of murine hepatocellular carcinoma cells, the inhibition of either IGF-2 or IGF-R1 significantly impaired the migratory and invasive capacities of these cells (Chen Y. W. et al., 2009). In addition, Yang et al. showed that IGF-2 knockdown suppressed the migration of human retinal microvascular ECs (HRECs) by approximately 90% (Yang et al., 2018). Furthermore, in a variety of ECs, including human uterine microvascular ECs, HUVECs, endothelial progenitor cells, and capillary ECs, 100 ng/mL IGF-2 stimulated migration by 50% to over 100% (Lee et al., 2000; Maeng et al., 2009; Volpert et al., 1996; Herr et al., 2003). Collectively, these findings reinforce the notion that IGF-2 serves as a potent migratory factor across various cell types. Interestingly, Leu27IGF-2’s limited effect on migration may be attributed to the relatively lower proportion of HMEC-1 expressing IGF-R2 compared to IGF-R1 and the IR. This suggests that the strong migratory effects observed with IGF-2 and Des(1-6)IGF-2 may be largely due to their interactions with IGF-R1 and IR, rather than reliance on IGF-R2 alone. Nonetheless, the migratory effects of Leu27IGF-2 observed in the transwell migration assay may be influenced by residual binding to IGF-R1, IR or both. While the manufacturer of the compound claims a 10–20 fold decrease in affinity for both IGF-R1 and IR, the original study described a 100-fold decrease in affinity for the IGF-R1 and no affinity for the IR for Leu27IGF-2 concentrations up to 200 ng/ml (Beukers et al., 1991). This inconsistency complicates interpretation, as it raises uncertainty about the degree to which observed effects are driven by IGF-2R-specific signaling versus partial cross-reactivity. Yet, variability in reported binding affinities of all variants makes it difficult to directly compare the effective concentrations of all IGF-2 variants. Therefore, assumptions regarding receptor involvement should be made with caution. Nevertheless, our findings suggest that Leu27IGF-2 interacts with IGF-R2 to some extent in promoting EC migration, challenging the notion of IGF-R2 as a decoy receptor. Supporting this, a study on human extravillous trophoblast cells showed that blocking IGF-R2 abolished IGF-2-induced migration, and that Leu27IGF-2 enhanced migration more effectively than IGF-2, suggesting IGF-R2 mediates these effects (McKinnon et al., 2001). Similarly, Maeng et al. demonstrated IGF-R2’s role in IGF-2-enhanced endothelial progenitor cell motility, with IGF-R2 blockade inhibiting migration, while IGF-R1 neutralization had no effect (Maeng et al., 2009). However, our results differ, as Leu27IGF-2 did not outperform IGF-2, but rather showed a weaker, more modest effect. Volpert et al. also investigated the effects of Leu27IGF-2 next to IGF-2 on the migration of capillary ECs. They found that 100 ng/mL Leu27IGF-2 was less effective than the same dose of IGF-2. However, at lower doses of 10–30 ng/mL, Leu27IGF-2 outperformed IGF-2, suggesting that lower concentrations may be more effective in stimulating migration, which further implies the involvement of IGF-R2 (Volpert et al., 1996).

Endothelial tube formation is key to driving the development of new blood vessels. This study reveals that IGF-2 and its variant, Des(1-6)IGF-2, markedly promote this process by increasing the number of nodes and total tube length, promoting endothelial cell-cell interactions and elongating vascular networks. Similar results were obtained by Lee et al. who showed that HUVECs form a capillary-like network on Matrigel upon stimulation with 100 ng/mL IGF-2 (Lee et al., 2000). Similarly, Yang et al. demonstrated that IGF-2 knockdown impairs tube formation with approximately 60%, but recombinant IGF-2 rescued the suppressive effects of microRNA-210 in HRECs, increasing tube formation by 160% (Yang et al., 2018). Sandovici et al. further confirmed the pro-angiogenic role of IGF-2, showing that it directly promotes tube formation in feto-placental ECs (FPECs) with increases in number of nodes (+80%), branches (+70%) and length (+60%) at 8 h. They also presented a significant stimulatory effect of Leu27IGF-2 on the tube formation capacity of FPECS though with more modest increases in number of nodes (+40%), branches (+30%) and length (+25%). Importantly, IGF-2’s effect was independent of IGF-1R, as inhibition of IGF-1R did not affect tube formation, suggesting IGF-2 acts through IGF-2R in FPECs, which lack IR expression (Sandovici et al., 2022). This contrasts our results where Leu27IGF-2 failed to significantly improve tube formation, implying no substantial involvement of IGF-R2 and making it impossible to rule out influence of IGF-R1 and IR in our study. It is essential to highlight that growth factor-reduced Matrigel was utilized in this study, which, despite lower concentrations, still retains bioactive molecules such as IGF-1, Transforming Growth Factor β, VEGF, Epidermal Growth Factor, Platelet-Derived Growth Factor, and nerve growth facto. A proteome array analysis of Matrigel revealed the presence of IGF-2, along with IGFBPs, including moderate amounts of IGFBP-6 (Talbot and Caperna, 2015). Residual amounts likely persist in growth factor-reduced Matrigel and may influence experimental outcomes; thus, caution is needed when interpreting results.

The role of IGFBPs in modulating IGF-2 activity was explored by examining the effect of IGFBP-6, a known inhibitor of IGF-2 (Bach, 2015b; Bach, 2016; Bach et al., 2013). IGFBP-6 was chosen for this study due to its distinct binding preference for IGF-2. Consistent with its known function, IGFBP-6 significantly attenuated IGF-2-induced migration in both the transwell migration assay and scratch wound healing assay, reducing migration levels to those observed in the negative control. A similar inhibitory effect was observed in the tube formation assay, reinforcing previous findings that IGFBP-6 suppresses IGF-2-driven actions including proliferation, survival, and differentiation across various cell types (Bach, 2015b; Bach, 2016; Bach et al., 2013). As expected, IGFBP-6 had no major impact on Des(1-6)IGF-2-induced migration and tube formation, indicating that Des(1-6)IGF-2 remains active despite the presence of IGFBP-6. This resistance to IGFBP-6 inhibition is due to the N-terminal truncation of Des(1-6)IGF-2, which reduces its affinity for IGFBPs while preserving its ability to bind the same receptors as IGF-2 (Francis et al., 1993). Interestingly, there is contradiction in the literature regarding the receptor-binding affinities of Des(1-6)IGF-2. Hashimoto et al. show that Des(1–6)IGF-2 binds IGF-R1 with an affinity equivalent to IGF-2, while its affinity for IR remains at ≥50% of that of IGF-2. Moreover, they also observed that Des(1-6)IGF-2 has a significantly greater affinity for IGF-R2 than IGF-2 (Hashimoto et al., 1995). In contrast, Luthi et al. found similar binding affinities for IGF-R1 between the two ligands but reported that Des(1-6)IGF-2 binds IGF-R2 with only 25% of IGF-2’s affinity (Luthi et al., 1992). Supporting this variability, Francis et al. demonstrated that Des(1-6)IGF-2 possesses approximately half the affinity of IGF-2 for both IR and IGF-R1, and only 20% for IGF-R2 (Francis et al., 1993). Notably, Gropep—the manufacturer of Des(1-6)IGF-2—states that the variant binds both IGF-R1 and IGF-R2 with affinities similar to wild-type IGF-2. In our experimental model using HMEC-1 cells, which predominantly express IGF-R1 and IR, both IGF-2 and Des(1-6)IGF-2 promoted EC migration and tube formation to a similar extent at equivalent doses in the absence of IGFBP-6. These findings suggest that, under our conditions, the affinities of IGF-2 and Des(1-6)IGF-2 for IR and IGF-R1 are comparable. Our findings support the hypothesis that IGFBP-6 acts as a key regulator of IGF-2 activity, but its inhibitory effect can be circumvented by structural modifications. Given that IGFBPs are naturally present in the human body (Allard and Duan, 2018), Des(1-6)IGF-2 may represent a more effective therapeutic target than IGF-2 for promoting angiogenesis. Besides its primary role of inhibiting IGF-2 activity, IGFBP-6 is also known to inhibit angiogenesis independently of IGF-2 (Zhang et al., 2012). As a result, the reduction in IGF-2 effects observed in our study may, in part, be attributed to the anti-angiogenic properties of IGFBP-6. However, when combined with Des(1-6)IGF-2, no significant decrease in effects was observed compared to Des(1-6)IGF-2 alone. This suggests that, under the conditions of our experiments, IGFBP-6 did not exhibit any inhibitory effects on its own, and the observed reduction in IGF-2 effects was due to the interaction between IGFBP-6 and IGF-2.

Given the pro-angiogenic effects of IGF-2 and Des(1-6)IGF-2 on migration and tube formation, we further assessed their impact on HMEC-1 secretome profiles. Both IGF-2 and Des(1-6)IGF-2 significantly increased IL-6, uPAR, and MCP-1 secretion; however, IGFBP-6 selectively attenuated this effect for IGF-2, but not Des(1-6)IGF-2. These findings further confirm that Des(1-6)IGF-2 can bypass IGFBP regulation, thereby sustaining its pro-angiogenic activity. To our knowledge, this is the first study linking IGF-2 to the secretion of these angiogenic factors. uPAR serves as the receptor for uPA and their interaction initiates the extracellular matrix degradation cascade (Wang et al., 2022). Upon activation, uPA converts plasminogen to plasmin, which subsequently activates matrix metalloproteases, facilitating ECM remodeling and clearing a path for cell migration (Quintero-Fabian et al., 2019; Zhai et al., 2022). Beyond its role in proteolysis, uPAR promotes angiogenesis by interacting with VEGFR2, enhancing its internalization and signaling to drive EC proliferation, survival, and migration (Herkenne et al., 2015). Additionally, uPAR forms a complex with α5β1-integrin, further supporting endothelial migration and vascular development (Uhrin and Breuss, 2013). Similarly, MCP-1 is a potent angiogenic factor. Salcedo et al. demonstrated that MCP-1 directly induces endothelial chemotaxis, an effect blocked by antibodies against MCP-1. *In vivo*, MCP-1 promoted blood vessel formation in the CAM assay (Salcedo et al., 2000). Furthermore, MCP-1 indirectly enhances angiogenesis by upregulating hypoxia-inducable factor 1 alpha, which in turn increases VEGF-A expression, amplifying the angiogenic response (Hong et al., 2005). IL-6 plays a multifaceted role in regulating inflammation, cell proliferation, differentiation, survival, immunomodulation, hematopoiesis, and tumorigenesis (Murakami et al., 2019; Middleton et al., 2014). While it is primarily known for its pro-inflammatory functions, studies have shown that IL-6 also promotes key angiogenic processes, including EC proliferation, migration, and tube formation (Fan et al., 2008; Gopinathan et al., 2015; Nilsson et al., 2005; Yao et al., 2006). Additionally, IL-6 enhances angiogenesis by upregulating VEGF expression in human cerebral and lymphatic ECs (Yao et al., 2006; Huang et al., 2016).

To extend our *in vitro* findings, we assessed the angiogenic effects of IGF-2 and its variants *in vivo* using the CAM assay. Our results demonstrated the angiogenic potential of IGF-2 and Des(1-6)IGF-2 in stimulating blood vessel formation *in vivo*. Both compounds significantly enhanced the number of blood vessels formed, while Leu27IGF-2 did not show a significant effect. A study by Herr et al. reported that a concentration of 25 μg/ml IGF-2 resulted in a notable increase in the vascularity index by approximately 225% (Herr et al., 2003). Similarly, Merckx et al. reported comparable findings, using 500 ng of IGF-2 to enhance blood vessel formation *in ovo*, resulting in a 50% increase in the number of blood vessels (Merckx et al., 2020). It is important to acknowledge that the CAM assay did not include co-administration of IGFBPs. The developing chicken embryo naturally produces endogenous IGFBPs, which regulate IGF bioavailability, stability, and receptor interactions (McMurtry et al., 1997; Allan et al., 2003). Additionally, IGF-2 is inherently present in the developing embryo, playing a crucial role in growth and development (McMurtry et al., 1997). Consequently, exogenous IGF-2 or its variants may interact with these endogenous factors, potentially confounding clear dose-response interpretations. While the CAM assay offered insight into the angiogenic effects of IGF-2 and Des(1-6)IGF-2, adult models (like the mouse Matrigel plug assay), which are less dependent on IGF-2 for growth, are needed to clarify their roles in post-developmental angiogenesis.

While our study highlights functional differences in angiogenic potential of IGF-2 variants, it does not examine downstream signaling pathways. As IGF signaling typically involves PI3K/Akt and MAPK/ERK activation (Bach, 2015a), future studies assessing the phosphorylation status of these components could offer mechanistic insight into variant-specific effects.

Overall, our findings underscore the distinct effects of IGF-2 and its variants on EC migration, tube formation, and *in ovo* angiogenesis. The robust responses observed with IGF-2 and Des(1-6)IGF-2, compared to the more modest effects of Leu27IGF-2, suggest that activation of IGF-1R and/or IR is central to promoting HMEC-1 motility and tube network formation. Still, Leu27IGF-2’s effects observed in the transwell migration assay point to a possible role for IGF-R2 in this process. Furthermore, the ability of IGFBP-6 to inhibit IGF-2-induced effects, but not those mediated by Des(1-6)IGF-2, highlights the critical role of IGFBP interactions in modulating IGF-2 bioavailability and activity. Together, these results offer new insights into IGF-2 signaling in vascular biology and potential therapeutic avenues. Of particular note, the enhanced potency of Des(1-6)IGF-2 suggests its potential as a more effective pro-angiogenic agent for therapeutic angiogenesis. However, the proliferative and potentially tumorigenic properties of IGF-2, which could potentially promote uncontrolled cell growth and metastasis, warrant caution when considering its clinical application (Belfiore et al., 2023; Brouwer-Visser and Huang, 2015; Livingstone, 2013). In contrast, elevating IGFBP-6 activity may serve to modulate IGF-2 signaling, offering therapeutic potential for conditions where angiogenesis inhibition is beneficial such as cancer or diabetic retinopathy. Future studies should assess the safety of Des(1-6)IGF-2 and explore IGFBP modulation to develop safer, more effective therapies.

## Data Availability

The datasets generated during and/or analysed during the current study are available from the corresponding author upon reasonable request.
